# Incidence of left ventricular thrombi in reperfused STEMI patients detected by contrast-enhanced CMR

**DOI:** 10.1186/1532-429X-17-S1-P164

**Published:** 2015-02-03

**Authors:** Heerajnarain Bulluck, Steven K White, Robert L Yellon, Shah Mohdnazri, Stefania Rosmini, Anish N Bhuva, Georg M Frohlich, Thomas A Treibel, Marianna Fontana, Amna Abdel-Gadir, Charlotte Manisty, Anna S Herrey, Reto A Gamma, Alex Sirker, James Moon, Derek J Hausenloy

**Affiliations:** The Heart Hospital/ University College London Hospital, London, UK; The Hatter Cardiovascular Institute, University College London, London, UK; The Essex Cardiothoracic Centre, Nethermayne, UK

## Background

Left ventricular (LV) thrombus formation remains a well-recognized complication following acute ST-segment elevation myocardial infarction (STEMI) in the primary percutaneous coronary intervention (PPCI) era, with potential devastating consequences such as embolic stroke. Echocardiography-based assessment of anterior STEMI patients, within the first 3 months of presentation, has reported an incidence of LV thrombi ranging from 8 to 15%. CMR not only provides higher resolution anatomical images but also has the ability for tissue characterization. Therefore, we hypothesize the true incidence of LV thrombi in reperfused STEMI patients using contrast-enhanced CE-CMR within one week would be more accurate.

## Methods

**Figure 1:**
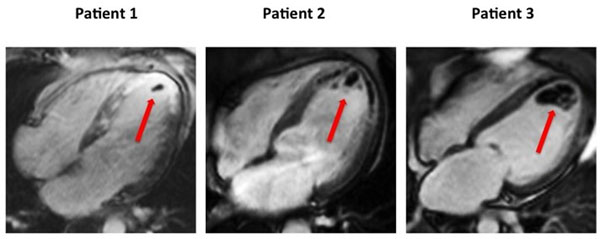
Early post contrast images of the 4 chamber views for 3 different patients. The red arrows show intra-cavity filling defects of different sizes of thrombi.

**Figure 2:**
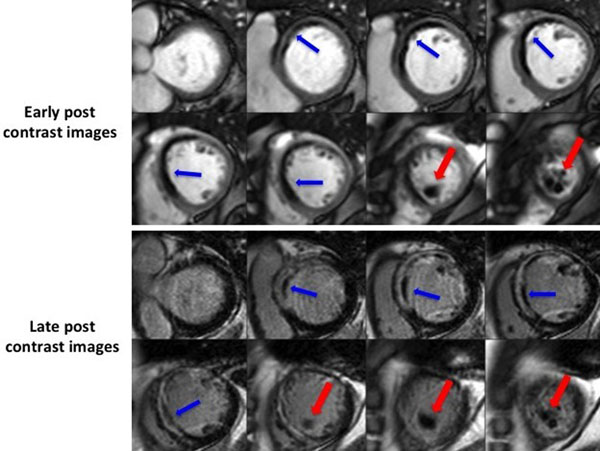
Short axis stack showing the differences between LV thrombus (red arrows) and MVO (blue arrows) during early and late post contrast images

139 PPCI-reperfused STEMI patients were recruited from two UK centres between July 2011 and September 2014. CE-CMR was performed at a median of 3 (2-5) days following admission [4 (2-5) days for anterior STEMI patients] on one of the 2 available scanners (1.5T Magnetom Siemens, Avanto and 3T Bio-graph mMR Siemens). Early post contrast images were acquired using a fixed long-inversion recovery time (TI) technique to null avascular tissue (Fig. 1). All cases of identified LV thrombi were diagnosed by an experienced investigator. To distinguish LV thrombi from areas of microvascular obstruction, the following previously described criteria were applied: (1) location (intra-cavity versus intra-myocardial); (2) contrast fill-in on subsequent late gadolinium enhanced images; and (3) differences in appearance (well-defined with sharp borders versus patchy and inhomogeneous) (Fig. 2).

## Results

All patients were successfully revascularized by PPCI. The majority of the patients were male (84%), the mean age was 59±11 years old, and the infarct related artery was as follows: LAD (50%), RCA (35%) and Cx (15%). Acute LV thrombi were identified in 19 out of the 139 patients, giving an overall incidence of 14% for all STEMI patients. All the LV thrombi were confined to patients presenting with an acute anterior STEMI (N=69 patients), and all were located at the apex, giving an incidence of 28% in anterior STEMI patients.

## Conclusions

CE-CMR at a median of 4 days detects a higher incidence of LV thrombi (28% of anterior STEMI patients) compared to previously reported studies using echocardiography (at a later time point). This is despite the use of anti-thrombotic therapy during PPCI, and subsequent dual anti-platelet therapy.

## Funding

This work was supported by the British Heart Foundation (FS/10/039/28270;FS/10/72/28568), the Rosetrees Trust, and the National Institute for Health Research University College London Hospitals Biomedical Research Centre.

